# Rapid Prediction of Moisture Content in Intact Green Coffee Beans Using Near Infrared Spectroscopy

**DOI:** 10.3390/foods6050038

**Published:** 2017-05-19

**Authors:** Adnan Adnan, Dieter von Hörsten, Elke Pawelzik, Daniel Mörlein

**Affiliations:** 1 Division Quality of Plant Products, Department of Crop Sciences, University of Goettingen, Carl-Sprengel-Weg 1, 37075 Goettingen, Germany; adnan.adnan@stud.uni-goettingen.de (A.A.); epawelz@gwdg.de (E.P.); 2 Institute for Application Techniques in Plant Protection, Julius Kühn Institute, Messeweg 11/12, 38140 Braunschweig, Germany; dieter.von-hoersten@jki.bund.de; 3 Department of Animal Sciences, University of Goettingen, Albrecht-Thaer-Weg 3, D-37075 Goettingen, Germany

**Keywords:** quality, rapid methods, infrared spectroscopy, *Coffea arabica* (Arabica), *Coffea canephora* (Robusta), chemometrics

## Abstract

Moisture content (MC) is one of the most important quality parameters of green coffee beans. Therefore, its fast and reliable measurement is necessary. This study evaluated the feasibility of near infrared (NIR) spectroscopy and chemometrics for rapid and non-destructive prediction of MC in intact green coffee beans of both *Coffea arabica* (Arabica) and *Coffea canephora* (Robusta) species. Diffuse reflectance (log 1/R) spectra of intact beans were acquired using a bench top Fourier transform NIR instrument. MC was determined gravimetrically according to The International Organization for Standardization (ISO) 6673. Samples were split into subsets for calibration (*n* = 64) and independent validation (*n* = 44). A three-component partial least squares regression (PLSR) model using raw NIR spectra yielded a root mean square error of prediction (RMSEP) of 0.80% MC; a four component PLSR model using scatter corrected spectra yielded a RMSEP of 0.57% MC. A simplified PLS model using seven selected wavelengths (1155, 1212, 1340, 1409, 1724, 1908, and 2249 nm) yielded a similar accuracy (RMSEP: 0.77% MC) which opens the possibility of creating cheaper NIR instruments. In conclusion, NIR diffuse reflectance spectroscopy appears to be suitable for rapid and reliable MC prediction in intact green coffee; no separate model for Arabica and Robusta species is needed.

## 1. Introduction

Moisture content (MC) is one of the most important quality parameters of green coffee beans. Most importing and exporting countries regulate MC as one of the quality standards for green coffee beans. The safety range for MC is 8.0–12.5%, based on fresh matter [1,2,3]. MC outside the safety range impairs the bean quality and safety. Beans with a MC above 12.5% are not allowed to be shipped and traded [4]. MC below 8% causes shrunken beans and an unwanted appearance [5], whereas MC above 12.5% facilitates fungal growth and mycotoxin production (e.g., ochratoxin A) that are risks to human health [6,7].

Coffee is harvested in the form of ripe berries and has a MC of more than 60% [8]. These ripe berries are processed through several steps of (wet or dry) postharvest treatments resulting in green coffee beans. Farmers generally dry the beans under the sun. The dried beans often do not meet the standard requirements for MC, resulting in a lower price [9]. For example, green beans obtained in the Bengkulu Province of Indonesia had a MC of 10.1–18.6% [10] and those in West Nusa Tenggara Province had a MC of 11.0–14.1% [11].

MC control is also important for the storability of the beans. An inappropriate storage environment (e.g., non-aerated silos and bag storage) affects MC fluctuation. The MC of green coffee beans stored in non-aerated silos increased up to 15.4% during rainy season. This moisture increase leads to the accumulation of glucose and an unpleasant taste in the beverage [12].

Furthermore, MC is crucial before the roasting process. The same roasting temperature and time with different MCs can result in different quality attributes—like color, density, and aroma—of the end product [13]. Consequently, an identical MC of green coffee beans is important for the roasting procedure in order to produce a consistent quality of roasted beans.

Therefore, a fast and accurate determination of MC in green coffee beans is vital. Up to date, the standard method for determining MC is the gravimetric method, where a drying chamber with a certain temperature and time is used to dry the beans and afterwards the mass loss is calculated. International standards for MC measurement of green coffee beans are The International Organization for Standardization (ISO) 1446, 1447, and 6673 [3,14]. Thereof, ISO 6673 which requires less preparation and the shortest drying time (105 °C for 16 h) is widely accepted as a reference method among importing and exporting countries. Apparently these gravimetric methods do not suffice when the information on MC is needed instantly [5] which is why we researched alternative methods.

Near infrared spectroscopy (NIRS) has been widely investigated for rapid, often non-destructive, determination of the compositional and quality traits of agricultural products. For example, previous work in our group predicted rapid and non-destructive analysis of mango quality attributes using NIRS and chemometrics [15]. NIRS makes use of the fact that near infrared (NIR) radiation in the range of 780–2500 nm predominantly interacts with hydrogen bonds—e.g., O–H, C–H, N–H, S–H. NIR radiation that hits a sample may be transmitted, absorbed, or reflected, this depends on the chemical composition and physical factors of the sample. The intensity of transmitted, absorbed, or reflected radiation is then recorded by NIRS [16,17]. 

Specific wavelengths (1450 and 1940 nm) were identified to be highly correlated with water content [3,18,19]. Predicting MC using NIRS in any agricultural product is more complex and should not be based on wavelengths limited to 1450 and 1940 nm. MC does not only reflect water, but also loss of volatile compounds during drying [3]. In fact, NIR has some disadvantages, e.g., overlapping of wavelengths that correspond to specific organic compounds, and scattering problems [16,20]. It is therefore necessary to carefully develop calibration models for NIR based predictions [18,19].

Prediction of MC by NIRS has been developed over years for many agricultural products [21]. A regression model was developed to predict MC in (ground) green coffee bean (*Coffea arabica* from Brazil) based on NIR diffuse reflectance (log 1/R) spectra [22]. To the best of our knowledge, this is the first study investigating the prediction of moisture content in intact green coffee beans of both *Coffea arabica* (Arabica) and *Coffea canephora* (Robusta) species by near infrared spectroscopy (NIRS) and chemometrics. The main goal of this paper was to study the feasibility of near infrared spectroscopy (NIRS) to predict moisture content (MC) in intact green coffee beans. We developed and validated calibration models based on diffuse reflectance spectra which were obtained using a benchtop near infrared instrument. Our decision to involve both Arabica and Robusta species stems from the fact that both species are commercially important but vary in their chemical composition. Furthermore, we used intact green beans such as no sample preparation would be needed—yet such an approach has not been documented. The results are especially relevant for those involved in coffee trading, production, and quality control. We also demonstrate the possibility of creating a simple NIR instrument which only uses a few important wavelengths to predict MC, rather than employing the full NIR spectrum.

## 2. Materials and Methods

A schematic representation of the experimental set up is given in the supplementary information (Figure S1).

### 2.1. Materials

Green Arabica and Robusta coffee beans that were harvested in 2013 were obtained from a local trading company in Indonesia. The materials were divided into separate sample sets for calibration and validation purposes (Table 1). The beans were placed in an open plastic box with the size of 15.5 × 11 × 6 cm, and were stored in a climatic chamber (Rumed^®^ type 1301, Rubarth Apparate GmbH, Laatzen, Germany) at 25 °C and a relative humidity range of 30–85%, in order to obtain a broad range of MC within 6–22%. Upon equilibration, samples were removed from the climatic chamber to record diffuse reflectance (log 1/R) data. Immediately thereafter, MC of the beans was determined.

### 2.2. Near-Infrared Spectroscopy

A bench top Fourier transform near infrared (FT-NIR) instrument with sample cup rotation (Thermo Nicolet Antaris MDS, Thermo Fisher, Waltham, MA, USA) was used to acquire diffuse reflectance spectra (log 1/R) of bulk samples of green coffee beans (40 g) on a Petri dish with a diameter of 7 cm.

Spectra were collected according to a workflow developed using the software Result Integration Software (Result^TM^ version 3.0, Thermo Fisher, Waltham, MA, USA). Internal background spectra were collected once every hour. High resolution diffuse reflectance (log 1/R) spectra at a wavelength range of 1000 to 2500 nm with 2 nm intervals were recorded as the averages of 64 scans. Thus, the spectra consisted of 1557 data points. Three replicates were acquired per sample and the spectra were averaged before further calculations. In total, this resulted in 108 spectra of 12 samples differing in moisture content, species, and origin.

### 2.3. Moisture Content Determination

MC (% wet basis) was determined was based on ISO 6673 [3]. A forced air electrical oven (Thermicon P^®^ type UT6120, Heraeus Instruments GmbH, Hanau, Germany) was used to dry approximately 10 g whole green coffee beans in open glass petri dishes (diameter: 14 cm, height: 2.3 cm) at 105 ± 1 °C for 16 h. Samples were limited to six origins with two replications per drying cycle in order to maintain an equal amount of mass loss during drying. The petri dishes were closed with glass lids immediately after drying had completed, and then they were stored in desiccators for 1 h in order to cool down the samples to ambient temperature. Finally, samples were weighted (Type LP 620 S, Sartorius AG, Göttingen, Germany) to calculate MC based on weight loss; data are given as the average from two replications (Equation (1)). Across all samples, average standard deviation of replicate MC determinations was 0.21% MC (Median: 0.08% MC).

(1) $$\mathrm{MC}=\frac{W_{w}-W_{d}}{W_{w}}$$

where MC is the moisture content (%) of green coffee beans (wet basis), W_W_ is the wet weight of the sample, and W_d_ is the weight of the sample after drying.

### 2.4. Data Processing

The statistical software (The Unscrambler^®^ X version 10.2 Network Client, CAMO software AS, Oslo, Norway) was used for further processing of the spectral data. Regression models to predict MC in green coffee beans were developed with a subset of calibration samples (*n* = 64), and then the models were tested using the subset of validation samples (*n* = 44) to evaluate the accuracy.

Firstly, spectral outliers were identified using Principal Component Analysis (PCA) and Hotelling’s T^2^ ellipse 5% plot, based on all samples’ (*n* = 108) raw spectra. Afterwards, several pre-processing methods were applied to compensate the disadvantages of NIR, e.g., the scattering and material size [16,23]. In detail, smoothing (moving average, Gaussian filter, median filter) window size of 3, 7, 11, 15, 19; Savitsky–Golay derivative (First derivative, two polynomial order; second derivative, two polynomial order; third derivative, three polynomial order) window size of 3, 7, 11, 15, 19; normalization (area, mean); baseline correction (baseline offset, linear baseline correction); standard normal variate (SNV); orthogonal signal correction (OSC) (non-linear iterative partial least squares algorithm, number of component 1); multiplicative scatter correction (MSC) (full MSC model); and extended multiplicative scatter correction (EMSC) were applied. Subsequently, the models were compared in terms of prediction accuracy and model robustness (number of latent variables). MSC and EMSC were applied to the calibration data. Upon model validation, the processing was also applied to the validation data set prior to prediction.

Calibration models were developed using both partial least squares regression (PLSR) and multiple linear regression (MLR). For PLSR, the full spectra (1557 wave numbers, mean centered) were used. Full cross validation was applied to estimate calibration errors. Regression coefficients were obtained from PLSR to determine the important wavelengths, i.e., those that correlated most to MC. A subset of selected wavelengths was then used as an input for full rank MLR and PLS regression to identify the most parsimonious yet robust model. Leverage correction was applied with MLR to estimate calibration errors. The calibration models derived from PLSR and MLR were evaluated by the number of latent variables (LVs), *R*^2^ of calibration, *R*^2^ of cross validation, root mean square error of calibration (RMSEC), and root mean square error of cross validation (RMSECV). Finally, all models were validated in terms of their prediction accuracy using a separate validation data set. Parameters used were *R*^2^ of prediction, root mean square error of prediction (RMSEP), standard error of prediction (SEP), bias, and residual predictive deviation (RPD) [22,24].

## 3. Results

### 3.1. Spectral Properties, Outliers, and Effect of Pre-Processing

**Figure 1:**
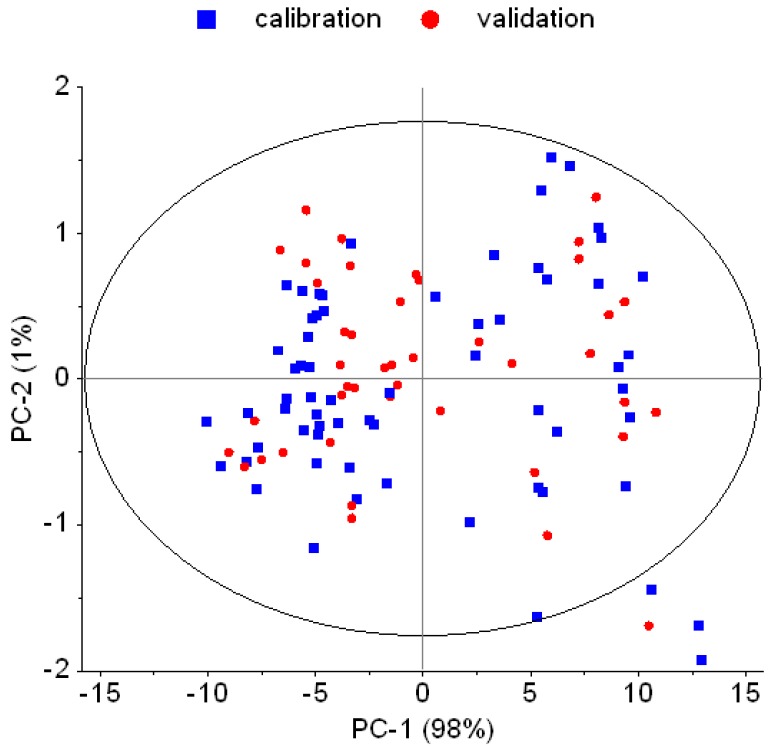
Score plot of principal component analysis (PCA) using raw infrared spectra (log 1/R) with Hotelling’s *T*^2^ ellipse for outlier inspection. Calibration samples (squares) and validation samples (circles) are marked accordingly. PC: principal component.

According to an initial PCA using all raw spectra and projection of the Hotelling’s T^2^ ellipse, four samples were suspected as spectral outliers (Figure 1). Subsequent modeling with and without these potential outliers, respectively revealed that model accuracy was not significantly affected. Thus, the suspected outliers were not excluded.

**Figure 2:**
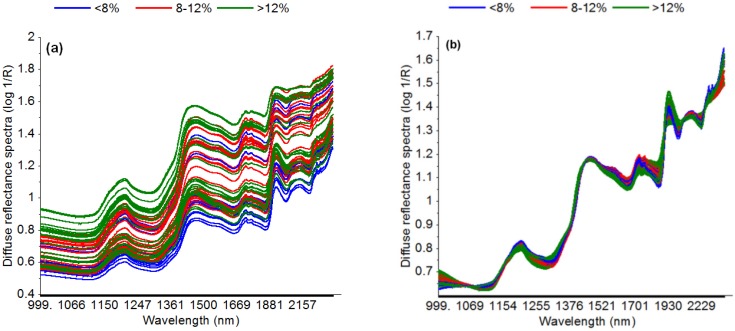
Diffuse reflectance spectra (log 1/R) of calibration model. Raw spectra (**a**); EMSC (extended multiplicative scatter) corrected spectra (**b**).

Inspection of the raw data also revealed that the NIR diffuse reflectance spectra of intact green coffee beans are influenced by scatter (Figure 2a). Several pre-processing methods were applied to eliminate the scatter. Application of EMSC proved to improve the prediction accuracy; the EMSC corrected spectra are shown in Figure 2b. Inspection of EMSC corrected spectra indicated that several wavelength regions reflect the chemical information regarding moisture content.

### 3.2. Prediction of Moisture Content from NIR Reflectance Spectra

Several preprocessing methods were applied to build the model (see Section 2.4). Nevertheless, none of the preprocessing methods yielded a better accuracy than models using raw data. Selected results of the various chemometric approaches to predict MC from NIR reflectance spectra are given in Table 2. The most parsimonious PLSR model on the full spectral range was achieved using raw spectra and three latent variables. Its prediction accuracy was, however, somewhat compromised when using the independent validation data set. Using the EMSC corrected spectra instead of the raw spectra yielded a similar *R*^2^ while the prediction errors were comparably low both for the calibration and the validation data set. Yet, this model used four latent variables, e.g., it was less parsimonious compared to the model based on raw data.

**Figure 3:**
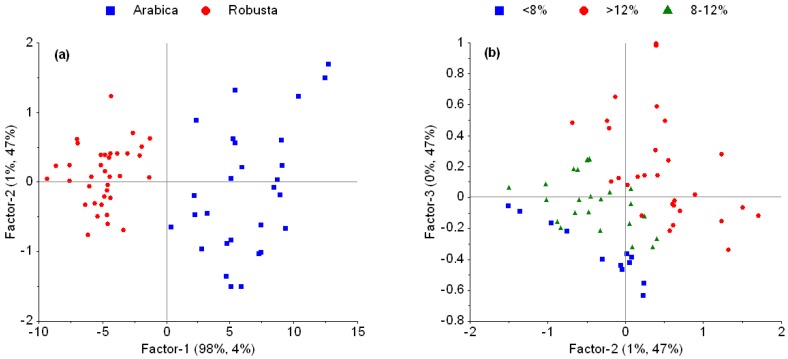

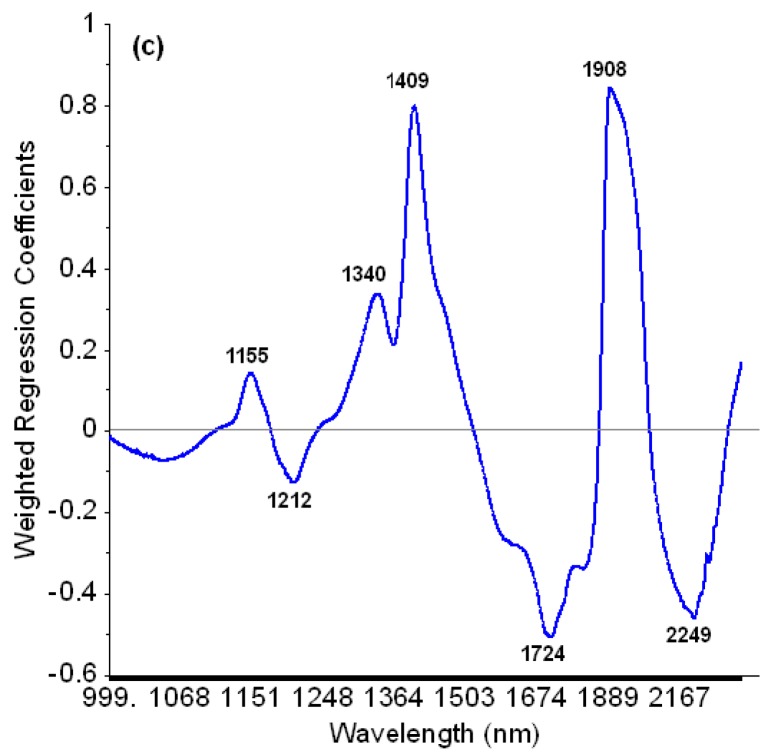
Score plots of PLSR for moisture content prediction based on raw diffuse reflectance (log 1/R) near infrared spectra. A distinct clustering of Arabica and Robusta coffee samples is observed when displaying PC 1 vs. PC2 (herein: factor-1 and factor-2) (**a**); Sample allocation is following moisture content indicating the importance of PC 2 and 3 for moisture prediction (**b**); Weighted regression coefficients obtained from PLSR using raw spectra (**c**).

Principal components (PCs) 1 and 2 of the PLSR model based on raw spectra explain 99% of spectral data variance and 51% of MC variance; a clear separation of Arabica and Robusta species is to be seen (Figure 3a). PC 2 and 3 together explain 94% of MC variance (Figure 3b).

Weighted regression coefficients obtained from PLSR on raw data (Figure 3c) were then used to study whether the model could be even simplified. Note that weighted and raw regression coefficients are the same as long as spectral data are not scaled but only mean centered; this was applied here. Seven wavelengths were selected due to their regression weights. That is, the intensities of 1155, 1212, 1340, 1409, 1724, 1908, and 2249 nm were used as input data to develop a MLR calibration model. Thus, a similarly accurate model was obtained (Table 2); the prediction error for the validation test set was significantly lower (*p* < 0.05) for the MLR model (0.93% MC) as compared to the EMSC model using raw data (0.57% MC). The resulting MLR model is given in Equation (2).

(2) MC (%)=−4.20+115.02 (V1)+ 0.40 (V2)– 116.18 (V3)+ 76.16 (V4)− 97.72 (V5)+ 63.76 (V6)− 17.59 (V7)

where, V1 to V7 are the intensities of the wavelengths 1155, 1212, 1340, 1409, 1724, 1908, and 2249 nm, respectively. When subjecting this spectral subset to PLS, the predictive ability of a three LV model was even improved as compared to the full-rank MLR model (Table 2); its prediction error (0.77% MC) was significantly lower than the MLR model (*p* = 0.015). It is, however, not significantly different from the PLSR model using raw data (*p* > 0.05).

**Figure 4:**
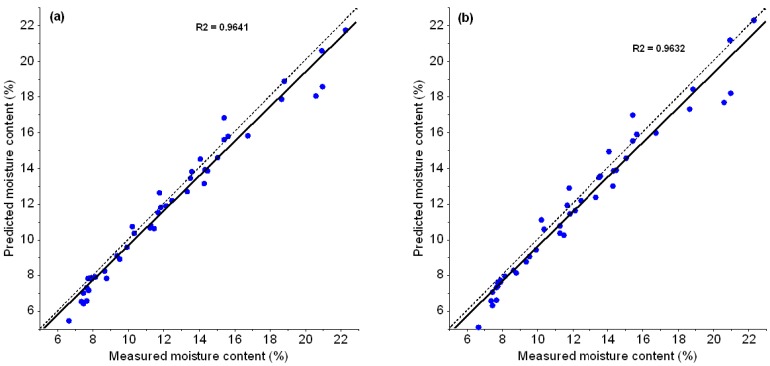
Predicted vs. measured moisture content of green coffee beans based on raw diffuse reflectance (log 1/R) near infrared spectra. (**a**) PLSR; (**b**) MLR.

PLSR and MLR using raw spectral data yielded a good correlation of reference versus predicted MC (Figure 4a,b). Also, the model’s bias is close to the error of the reference method (0.21% MC, see 2.3.).

## 4. Discussion

### 4.1. Outliers and Effect of Pre-Processing

For outlier detection, PCA and subjection of the Hotelling’s T^2^ ellipse along with residuals and influence plot, and Q-residuals plot, were used which are common approaches in multivariate analysis. Identifying true outliers is important to prevent false inferences [25]. In this experiment, four samples were suspected to be outliers (Figure 1). Explained spectral variance (PC1 + PC2) based on diffuse raw data reflectance (log 1/R) was 99%. Elimination of suspected outliers did not increase the explained variance. Further comparisons of PLSR with and without the suspected outliers yielded only very slight improvement in *R*^2^ which indicates that the suspected outliers were no real outliers. Similarly, Morales-Medina and Guzmán [26] examined multivariate data using Hotelling’s T^2^ ellipse. They also decided to not exclude the suspected outliers because they did not significantly affect the explained variance found through PCA.

Various pre-processing methods were applied to the raw spectra. This aims at reducing noise and improving the accuracy of the prediction model [27]. EMSC was effective to remove scatter which was shown also in other studies [28]. Accordingly, the prediction errors were the lowest when using EMSC corrected data for PLSR (Table 2). The resulting model, however, was surprisingly less parsimonious, i.e., it needed one more latent variable. Pizarro et al. [27] also reported that none of the pre-processing methods studied (first and second derivation, MSC, standard normal variate) improved the prediction for ash and lipid content in roasted coffee significantly as compared to using raw data; only OSC and direct orthogonal signal correction (DOSC) enhanced the model performance remarkably. 

### 4.2. Prediction of Moisture Content Using NIR Infrared Spectra

Raw spectra were selected as an input to build the final PLSR model because this resulted in the lowest number of latent variables, the highest *R*^2^ and lowest root mean square error compared to other pre-processing methods (Table 2). A model with these criteria is preferable. Kamruzzaman et al. [29] also considered the number of latent variables together with *R*^2^ and prediction errors to select the most appropriate model for prediction of water, fat, and protein content in lamb meat. Both the robustness and the predictive ability of a given model are of importance. If one considers only *R*^2^, RMSEP, or RPD, which reflect the predictive ability, likely models using more latent variables would be preferred over models using less latent variables. In terms of robustness, however, a model using less latent variables is less prone to overfitting than a model using more latent variables. 

Further examination of the PLSR score plots (based on raw spectra) revealed a distinct clustering of Arabica and Robusta samples on the first latent variable, explaining 98% in the spectral data variance but only 4% of moisture variance (Figure 3a). To understand this clustering, the loading weights of the first LV were inspected. As a result, important wavelengths are related to several chemical compounds, e.g., caffeine, chlorogenic acid, lipids, protein and amino acids, sucrose, carbohydrates, trigonelline and, of course, water [30]. These compounds were shown to vary between species which is why their spectral contributions can be used to discriminate between species [31,32] Using PC 2 and 3 which together explain 94% of moisture variance, samples are allocated according to moisture content levels (Figure 3b). Thus, a three component PLSR model allows prediction of moisture content on both Arabica and Robusta species. The advantages of inputting raw spectra rather than pre-processed spectra firstly reduces the complexity of calculations and therefore secondly reduces the computation time. These advantages will be useful for online and real time prediction in the future.

The statistical parameters of calibration and prediction accuracy were similar for the developed PLSR models, especially for the model based on EMSC corrected spectra. This indicates that the PLSR model is robust in terms of predicting unknown samples accurately. We also investigated PLSR models based on raw spectra within individual species. However, the results were not better than the PLSR model which was developed across species. The PLSR model obtained in this experiment resulted in a similar accuracy compared to what was reported by Morgano et al. [22]. That study predicted the MC of green Arabica coffee beans, based on smoothed spectra, which yielded an *R*^2^ of calibration = 0.507, *R*^2^ of validation = 0.669, and RMSEV of 0.55% MC (*R*^2^ recalculated from r).

Even simplified MLR and PLS models were built using selected wavelengths based on their relative importance in the PLSR model. This experiment showed that near infrared diffuse reflectance intensities at 1155, 1212, 1340, 1409, 1724, 1908, and 2249 nm highly correspond to MC (Figure 3c). According to Ribeiro et al. [30], these wavelengths are related to the absorbance of the second overtone of C–H, first combination overtone of C–H, first overtone of O–H and N–H, second overtone of C=O, and combination of O–H and N–H, respectively. Obviously, these wavelengths are not exactly located at the water bands which indicate that it may well be useful to apply indirect relationships in prediction models. Plus, it was shown that the degradation of organic components during drying for MC determination needs to be considered. Reh et al. [3] proved that, using ISO 6673, the beans lose 0.39% of their mass besides water. Thus, MC is calculated as a sum of extracted water and mass losses of other compounds. Similarly, Pan et al. [33] found that MC in beet slices highly corresponded to spectral intensities at 968, 1078, and 1272 nm, i.e., not exactly located at the water bands.

The MLR model, as well as the PLS model based on the spectral subset, yielded a good accuracy both for calibration and validation thus proving their robustness (Figure 4b). The biases measured by PLSR and MLR were close to the method error of determining moisture content based on ISO 6673. Moreover, the ratio of the standard deviation of the target variable and the SEP of a given model, commonly referred to as RPD (residual predictive deviation), is often used to assess the performance of prediction models; higher RPD values indicate a better predictive performance [24]. Here, the models yielded RPD values of about 3 to 8 (Table 2) which is considered good [34]. This shows the potential of near infrared spectroscopy to replace the reference method when a fast and non-destructive prediction is needed, e.g., when trading or for in-line process control.

Finally, the remarkable reduction of variables without a relevant loss of accuracy opens the possibility of creating a simple NIR instrument which only uses a few important wavelengths to predict MC, rather than employing the full NIR spectrum. Specific LED light sources emitting only selected wavelengths can potentially reduce the costs of an NIR instrument.

## 5. Conclusions

The results indicate that a fast, non-destructive prediction of moisture content in intact green coffee beans is feasible using near infrared diffuse reflectance spectroscopy. EMSC effectively reduces scatter apparent in raw spectra. Thus, the prediction accuracy using EMSC corrected spectra is improved at the cost of a somewhat less parsimonious model. A simplified model based on only seven selected wavelengths points to the possibility of a cheaper instrumentation. The calibration model can be applied for both Arabica and Robusta species. In conclusion, NIR is deemed feasible to replace gravimetric methods for routine applications where a timely result may outweigh the loss of accuracy as compared to the drying methods.

## Figures and Tables

**Table 1:** Characteristics of the coffee samples including species and origin.

No.	Purpose	Species	Origin
1	Calibration	Arabica	West Nusa Tenggara
2			South Sulawesi
3			Aceh
4		Robusta	South Sumatera
5			Bali
6			East Java
7			North Sumatera
8	Validation	Arabica	West Java
9			North Sumatera
10		Robusta	South Sumatera
11			East Java
12			Bengkulu

**Table 2:** Statistical parameters of the developed prediction models for moisture content (MC) in green coffee beans using near infrared spectra.

Model	Parameter	Full Spectral Range PLSR		Spectral Subset	
		Raw	EMSC	Raw (MLR)	Raw (PLS)
Calibration	LVs	3	4	n/a	3
	*R*^2^ calibration	0.9834	0.9850	0.9839	0.9743
	*R*^2^ cross validation	0.9802	0.9811	0.9779	0.9698
	RMSEC (% MC)	0.52	0.49	0.51	0.65
	RMSECV (% MC)	0.58	0.56	0.60	0.71
Prediction	*R*^2^ prediction	0.9641	0.9817	0.9632	0.9669
	RMSEP (% MC)	0.80	0.57	0.93	0.77
	Bias (% MC)	0.42	0.28	0.45	0.39
	RPD	6.21	8.53	3.47	6.39

PLSR: partial least squares regression using full spectral range (1000 to 2500 nm, 1557 data points); MLR/PLS: multiple linear and partial least squares regression using selected wavenumbers (1155, 1212, 1340, 1409, 1724, 1908, and 2249 nm); LVs: Latent variables (for PLS only); *R*^2^: the coefficient of determination; RMSEC: root mean square error of valibration; RMSECV: root mean square error of cross validation; RMSEP: root mean square error of prediction; SEP: standard error of prediction; RPD: residual predictive deviation; n/a: not applicable; MC: moisture content.

## Supplementary Materials

The following are available online at www.mdpi.com/2304-8158/6/38/s1, Figure S1: Schematic representation of the experimental set up.
