# Supplementary figures and images for: Rapid Prediction of Moisture Content in Intact Green Coffee Beans Using Near Infrared Spectroscopy
Source: Foods. 2017 May 19;6(5):38. doi: 10.3390/foods6050038 (PMC5447914; doi:10.3390/foods6050038)

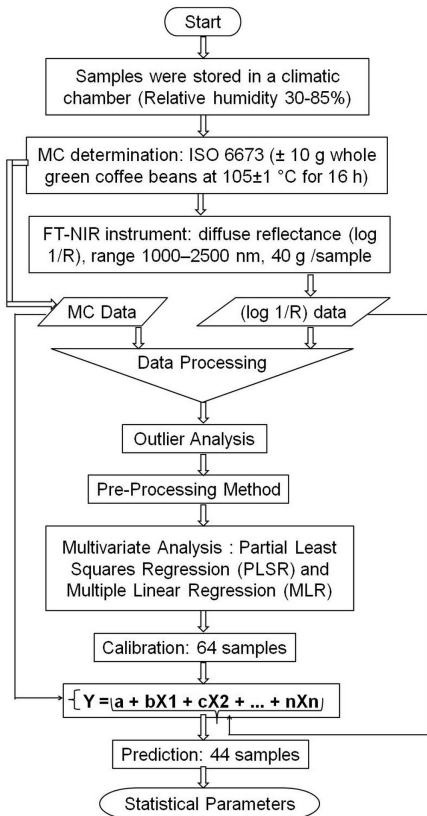

Supplement: Supplementary file 1 [file foods-06-00038-s001.pdf]
